# Erythema multiforme: integrating the superfluous diagnosis to a veracious conclusion (case report)

**DOI:** 10.11604/pamj.2020.37.129.25295

**Published:** 2020-10-06

**Authors:** Inukonda Lakshmi Mounica, Katne Tejaswi, Tirumala Chennaiah, Janumpally Varshitha Thanmai

**Affiliations:** 1 Department of Oral Medicine and Radiology, SVS Institute of Dental Sciences, Mahabubnagar-509002, Telangana, India

**Keywords:** Herpes simplex virus, drug induced, erythema multiforme, pathogenesis

## Abstract

Erythema multiforme (EM) is an acute, self-limiting hypersensitivity reaction that ranges from a mild cutaneous, exanthematous variant with minimal oral involvement to a progressive fulminating severe variant with extensive mucocutaneous epithelial necrosis (SJS & TEN). Knowing the etiological spectrum is the key for management in EM where sometimes leads to mortality. There are no specific diagnostic tests for EM, diagnosis is mainly clinically supported. Here in we report series of cases of EM with different etiological spectrum.

## Introduction

EM is an acute, recurrent, self-limited, inflammatory mucocutaneous disease that manifests on the skin and often on the oral mucosa which occasionally can give rise to systemic upset and possibly compromise life [1]. The skin lesions may take several forms such as macules, papules, vesicles, bullae, and hence the term “multiforme”. The classic skin lesion is it consists of central blister or necrosis with concentric rings of variable color named typical “target” or “iris” lesion which is the pathognomonic feature of EM. Exact etiology is not known, but it is said to be a hypersensitivity reaction. EM has been classified into 2 subgroups, minor and major forms. EM minor (EMm) is mainly a cutaneous disease, mucosal involvement is uncommon and EM major (EMM) typically involves two or more mucous membranes [2].

## Patient and observation

**Figure 1:**
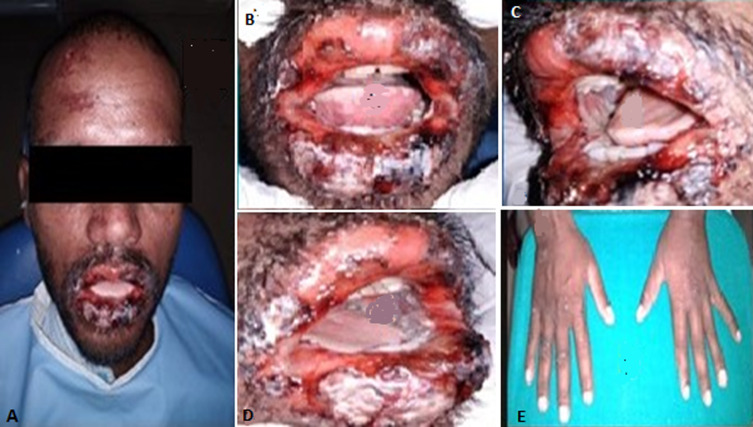
series of pictures showing target lesions on extremities (A, B), multiple encrustations, ulcerations with clinically evident bleeding (C, D, E)

**Figure 2:**
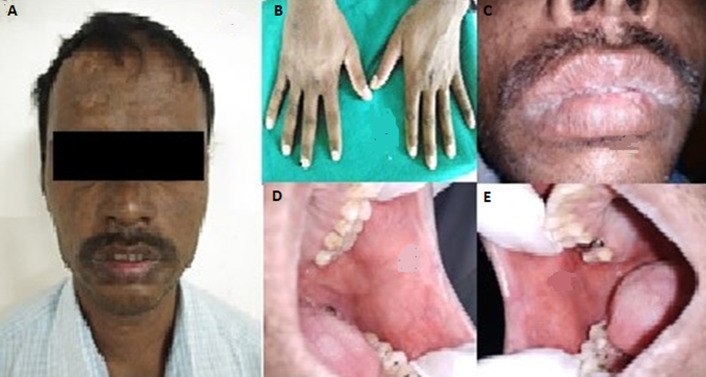
post-operative pictures showing complete regression of the lesions

**Case 1:** a 24-year-old male patient reported with a complaint of extensive painful ulcers and difficulty in eating since 5 days. Significant history of throat infection and common cold two weeks back for which he took azithromycin and diclofenac sodium prescribed by the local private practitioner, subsequently he developed irregular ulceration and hemorrhagic crust on the lips (Figure 1 A) extraorally. Intraorally, diffuse erosions, irregular ulcerations were noticed on bilateral buccal mucosa, tongue (Figure 1 B, C, D). A diagnostically significant finding was the presence of multiple concentric target or iris lesions on palmar surfaces (Figure 1 E). Sudden onset of lesions after the positive drug history associated with aggressive clinical features made us to give the diagnosis as drug induced EM. Patient was advised to discontinue the medication and was commenced with tab Prednisone 30mg b.d along with benzydamine hydrochloride (tantum) mouthwash and evaluated after a week (Figure 2).

**Figure 3:**
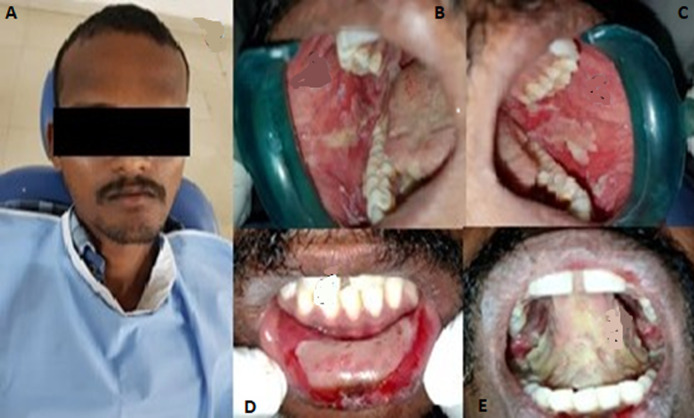
pre-operative pictures showing diffuse erosions and encrustations were noticed on bilateral buccal mucosa, lower labial mucosa and tongue

**Figure 4:**
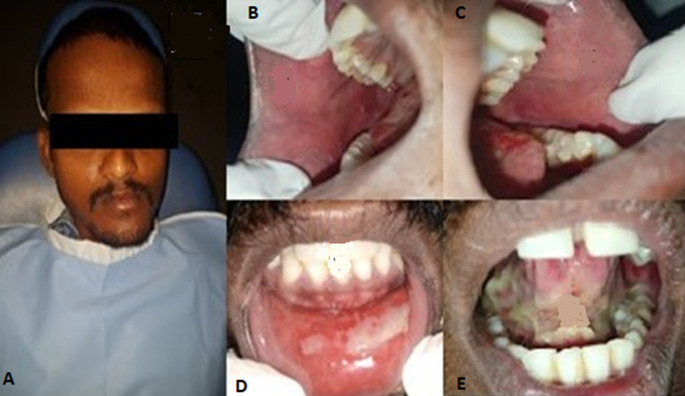
complete regression of lesions of bilateral buccal mucosa and partial regression on lower labial mucosa and tongue was noticed

**Case 2:** a 21-year-old male patient reported to our department with the complaint of ulceration in the mouth since 5 days. Patient had fever, cold and diarrhoea 10 days back following which he noticed vesicles and then ruptured in the mouth which made difficulty to consume foods. Intraorally encrustations, diffuse multiple ulcers, and erosions were noticed on the lower labial mucosa, bilateral buccal mucosa and tongue which were covered by pseudo-membrane, bleeding on provocation was present and were tender on palpation (Figure 3). To further investigate, herpes simplex virus (HSV) antibody titre was done, HSV 2 IgG was positive. Although IgG indicates the past infection, based on the antibody titre and acceptable history made us to give the diagnosis of HSV 2 induced EM. The patient was commenced with tab Acyclovir 400mg t.i.d., along with tantum mouthwash for a week and evaluated after a week (Figure 4).

## Discussion

Dating back to 1817, EM was first recognized by Bateman and Bulkley. Later, Hebra, in 1866 described the morphologic features of the eruption under the term “erythema exsudativum multiforme” [1] as an acute mucocutaneous hypersensitivity reaction characterized by skin eruptions that causes a variety of skin lesions-hence named ´multiforme´ with or without mucous membrane lesions [2]. Etiology spectrum is variable which include drugs (NSAIDs, sulphonamides, phenytoin, and carbamazepine), infectious agents and food additives. Mostly it is predominated by HSV in 70% to 80% of cases. Prevalence varies from 35% to 65% [3]. Exact pathogenesis is still unknown, but available literature suggests, it is a T-cell-mediated immune reaction to the precipitating aetiological agent leading to immune mediated epidermal damage causing vesicles, blisters and erosions. In HSV and drug associated EM it occurs due to the production of interferon-γ (IFNγ) and tumor necrosis factor α (TNF- α) respectively [4]. In recurrent EM HLA DQ3 is helpful in distinguishing HAEM from other EM-like lesions [4]. EM occurs within 20-40 years with male preponderance in consonance with present cases. It presents from mild limited disease to a severe, wide-spread and life-threatening illness [5]. It is classified into 2 subgroups, minor and major, minor involves less than 10% of the body surface area, predilection for the extensor surfaces of the extremities. Mucosal involvement is uncommon, when present, only one site is affected. Major typically involves two or more mucous membranes with more variable skin involvement [6]. Less than 10% of the body surface area but more severe than EMm. Oral lesions are usually widespread and severe [3]. The hallmark of EM is presence of necrotic tissue tags, blood encrustations and typical “target” lesions [6]. Clinical differential diagnosis included herpetic gingivostomatitis, mucous membrane pemphigoid and pemphigus vulgaris can be given for the above present cases.

There is no specific diagnostic test for EM. Diagnosis is purely based on the history and classical presentation itself. Viral research is carried out in viral etiology [2]. Biopsies are advised only in the early vesicular lesions and not in the ulcerated ones as histopathologic appearances are nonspecific. Immunostaining shows perivascularly, non-specific immune deposits of IgM, C3, and fibrin [7]. To differentiate herpes-associated EM from drug-associated EM and SJS, the detection of intralesional HSV-DNA via polymerase chain reaction, as well as immunohistochemistry for IFNγ and TNFα, may be useful tests. A rising antibody titre between the acute and convalescent phases of EM major/ SJS may confirm M. pneumonia infection [4]. Management is of utmost important. Precipitants which are responsible should be removed out along with supportive care by topical analgesics, anaesthetics, soothening mouth rinses and soft diet. Corticosteroids remains the mainstay in management along with antiviral drugs in order to deny HSV infection. Recurrences are seen in less than 5% of cases [8]. Although being a relatively common condition in reproductive aged women, there is a dearth of data regarding EM in pregnancy [9]. EM is rare in children < 3 years and > 50 years with male predominance; whereas SJS/TEN occurs equally in both sexes with predominance in older patients [10].

**Key messages:** 1) EM is a hypersentivity reaction. 2) 80% of the cases have HSV (herpes simplex virus) superimposition. 3) Pathagnomic feature of EM - necrotic tissue tags, blood encrustations, typical “target” lesions. 4) Has inclination towards adults, rare in children and elderly people. 5) No specific diagnostic tests, clinical presentation itself gives the diagnosis. 6) Sometimes, clinical features are superimposed with other etiological features, where the proper diagnosis plays vital role in the management.

## Conclusion

EM as known by its hypersensitivity reaction, it can be precipitated by any of its etiological agents. Recognition and removal of the aetiological agent is crucial step in the management of EM. Early diagnosis remains essential to promptly initiate appropriate management and proper follow up.
